# TMIGD1 Inhibited Abdominal Adhesion Formation by Alleviating Oxidative Stress in the Mitochondria of Peritoneal Mesothelial Cells

**DOI:** 10.1155/2021/9993704

**Published:** 2021-08-14

**Authors:** Yunhua Wu, Enmeng Li, Zijun Wang, Tianli Shen, Cong Shen, Dong Liu, Qiuying Gao, Xuqi Li, Guangbing Wei

**Affiliations:** ^1^Department of General Surgery, The First Affiliated Hospital of Xian Jiaotong University, Xian, 710061 Shaanxi, China; ^2^Department of General Surgery, Shaanxi Provincial People's Hospital, Xi'an, 710061 Shaanxi, China; ^3^Department of Radiology, The First Affiliated Hospital of Xi'an Jiaotong University, Xi'an, 710061 Shaanxi, China; ^4^Department of Haematology, Shaanxi Provincial People's Hospital, Xi'an, 710061 Shaanxi, China; ^5^Department of Talent Highland, The First Affiliated Hospital of Xian Jiaotong University, Xian, 710061 Shaanxi, China

## Abstract

**Background:**

Postoperative abdominal adhesion remains one of the frequent complications after abdominal surgery and lacks effective intervention. Peritoneal mesothelial cell injury and healing play crucial roles in the process of adhesion formation, and identifying this mechanism might provide new insight into possible new therapeutic strategies for this disease. Transmembrane and immunoglobulin domain-containing 1 (TMIGD1) has been proven to protect renal epithelial cells from injury induced by oxidative stress and has also been identified as a novel adhesion molecule. Here, we investigated the role of TMIGD1 and its possible mechanism in adhesion formation.

**Materials and Methods:**

Immunohistochemistry (IHC), qPCR, and immunofluorescence (IHF) were used to detect the expression of TMIGD1. The grade and tenacity score of adhesion were used to evaluate the adhesion formation conditions. A TMIGD1-overexpressing HMrSV5 cell line was established. MTT assay, Western blotting, Annexin V apoptosis analysis, and CK19 staining were used to measure mesothelial cell viability, apoptosis, and completeness. ROS and MDA detection were used to measure mesothelial cell oxidative stress levels. JC-1 staining, IHF, and transmission electron microscopy were performed to assess mitochondrial function. Scratch-wound and adhesion assays were used to evaluate the adhesion ability of mesothelial cells.

**Results:**

First, we showed that TMIGD1 was decreased in mouse abdominal adhesion tissue and peritoneal mesothelial cells. Second, TMIGD1 overexpression inhibited adhesion formation. Third, TMIGD1 overexpression protected mesothelial cells from hydrogen peroxide- (H_2_O_2_-) induced oxidative stress injury. Fourth, TMIGD1 overexpression alleviated oxidative stress by protecting the mitochondrial function of mesothelial cells. In addition, TMIGD1 overexpression enhanced mesothelial cell adhesion.

**Conclusion:**

Our findings suggest that TMIGD1 protects mesothelial cells from oxidative stress injury by protecting their mitochondrial function, which is decreased in regular abdominal adhesion tissue. In addition, TMIGD1 enhances peritoneal mesothelial cell adhesion to promote healing.

## 1. Background

Postoperative abdominal adhesion remains one of the most frequent complications after abdominal surgery, which occurs in approximately 67-93% of patients undergoing abdominal operations [1]. It further leads to intestinal obstruction, chronic abdominal pain, female infertility, and many other complications [2]. However, there is no effective management strategy for postoperative abdominal adhesion other than enterolysis, which is an invasive method with a high probability of reinjury and readhesion [3]. Although it brings about a large number of serious health problems, multifarious innovations attempted to prevent postoperative abdominal adhesion have so far failed to take effect.

Adhesion formation is a complicated pathophysiologic process that involves the inflammatory response, fibrosis, and mesothelial cell healing [4]. The mesothelium is a monolayer located on the peritoneum that forms a smooth peritoneal surface [5]. After abdominal injury or trauma, the inflammatory response is activated, followed by the formation of fibrosis, and the injured mesothelia cells heal within 7-10 days after the surgical procedure [6]. Mesothelial cell plays a critical role in the process of peritoneal tissue healing and adhesion formation. Mesothelial cell damage, loss, and epithelial-mesenchymal transition might be causes of abdominal adhesion following peritoneal damage [7]. In addition, adhesion could be effectively alleviated if some measures are used to enhance the regeneration ability of mesothelial cells and to rebuild the intact mesothelial cell layer in the early stage of abdominal adhesion [8]. Nevertheless, the underlying mechanism is elusive at present.

Oxidative stress is defined as a disrupted balance between oxidative molecules and inadequate antioxidant defense mechanisms [9]. Although the severity varies, it occurs in almost all surgery, such as endoscopic and Lichtenstein hernia repair [10]. Oxidative stress generates reactive oxygen species (ROS), which are highly destructive to cellular functions; it not only has a direct cytotoxic effect on mesothelial cells but also induces apoptosis of mesothelial cells [11]. Both mechanisms create a further injury of the mesothelial cells lining in the abdominal cavity beyond the injury created by surgical manipulation and enhance the probability of creating postoperative adhesions. The major intracellular sources of ROS are the electron transport chain in the mitochondria [12]. Some articles pointed out that hypoxia alters mitochondrial fusion and fission and oxidative phosphorylation, which causes overproduction of ROS by remodeling the electron transport chain [13].

As a family member of IGPR-1, transmembrane and immunoglobulin domain-containing 1 (TMIGD1) has been identified to be a novel cell adhesion molecule. Recent studies have demonstrated that TMIGD1 protects epithelial cells from oxidative injury. Downregulation of TMIGD1 is associated with increased peritoneal damage after oxidative stress induced by ischemia and reperfusion in mice [14]. These findings suggest a protective role of TMIGD1 against oxidative stress in the process of adhesion formation, but the underlying mechanism remains unclear. TMIGD1 also regulates the expression of p21Cip1/p27Kip1 in some renal cancers, acting as a tumor suppressor [15]. This study tested the hypothesis that TMIGD1 protects mesothelial cells from oxidative stress injury by protecting their mitochondrial function and enhances peritoneal mesothelial cell adhesion to promote healing, whose expression is suppressed in abdominal adhesion formation. Our findings support TMIGD1 as a therapeutic target to prevent postoperative abdominal adhesion, and TMIGD1 upregulating agents could be used during surgery to prevent adhesion.

## 2. Materials and Methods

### 2.1. Model of Adhesion

A C57BL/6 mouse abdominal adhesion model was established as previously described [16]. Twenty mice were randomly divided into two groups. After the mice were anesthetized (25% *W*/*V* isoflurane in propylene glycol) and sterilized, a 1 cm long incision was made in the central part of the mouse abdomen. The lower right cecum and the adjacent abdominal wall were scraped to the needle-like hemorrhagic spots to establish the postoperative abdominal adhesion model. The cecum was then placed in the abdominal cavity adjacent to the damaged abdominal wall, and the abdominal cavity was finally closed. Animals were sacrificed at 3 or 10 days following the procedure, and then two independent researchers assessed the score of adhesion. Then, tissue specimens were collected, including abdominal adhesion tissue and the surrounding normal bowel wall of the cecum and right-side abdominal wall (used as normal peritoneal tissue). The adhesion score system was as reported previously [17].

### 2.2. Cell Culture and Establishment of the TMIGD1-Overexpressing Cell Line

The human mesothelial cell line HMrSV5 was purchased from the Shanghai Institute of Cell Biology, Chinese Academy of Sciences, and cultured in minimum essential medium (Gibco, Thermo Fisher Scientific, Beijing, China) supplemented with 10% FBS (Gibco BRL, Carlsbad, CA, USA) at 37°C and 5% CO_2_. The overexpression vectors for humans were purchased from GenePharma Co., Ltd. (Shanghai, China). The transfection was implemented in accordance with the manufacturer's guidelines.

### 2.3. Lentivirus Transfection

The transfection of TMIGD1-overexpression lentivirus in vivo was as follows. Twenty-four mice were randomly split into three groups: the sham group only underwent open and close operations, and the other two groups underwent abdominal adhesion model operations as described above. After establishment of the abdominal adhesion model, 50 *μ*L 1 × 10^9^ lentivirus was spared around the injured peritoneal tissue, and then the injured cecum was placed adjacent to the injured abdominal wall. Ten days after the operation, the mice were sacrificed, and the expression of TMIGD1 in the peritoneal tissue of TMIGD1-overexpressing mice was tested by IHC and IHF to prove that the construction was successful. Adhesion tissue was collected and divided into two parts: one part was stored at -80°C, and the other part was fixed with 10% paraformaldehyde.

### 2.4. Immunohistochemistry (IHC) and Immunofluorescence (IHF) Staining

First, tissues collected from the abdominal cavity of mice were immersed in 10% formol for 24 hours. Next, 4 *μ*m thick paraffin sections were obtained, and IHF and IHC were performed according to the manufacturer's instructions. Sections were incubated with primary antibodies against TMIGD1 (1 : 100, Bioss, Beijing, China), HSP60 (1 : 500, Wuhan Servicebio Technology Co., Ltd., China), and CK19 (1 : 50, Wuhan Google Biotechnology Co., Ltd., Wuhan, China) over one night at 4°C. A series of pictures were taken using a Nikon Eclipse C1 confocal laser scanning microscope (Nikon Corporation, Tokyo, Japan). The assessment IHC was performed as reported previously [18], and at least five fields were selected for every section. The scoring system was as follows: a score of 0 indicated tissues with no expression, a score of 1 indicated tissue with weakly positive expression, a score of 2 indicated tissues with positive expression, a score of 3 indicated tissues with strongly positive expression, and a score of 4 indicated tissues with extremely abundant expression. For the evaluation of the expression of TMIGD1 in the IHF, we randomly selected at least ten fields for each section and measured the related green marked TMIGD1 or red marked HSP60 in CK19-marked mesothelial cells.

### 2.5. Western Blotting

Western blotting was performed, following that reported in some studies [19]. A RIPA Protein Extraction Kit (HeTe, Xi'an, Shaanxi, China) was used to extract protein from mesothelial cells. A 12% sodium dodecyl sulfate polyacrylamide gel was used for electrophoresis. The primary antibodies, including anti-TMIGD1 (Bioss, Beijing, China, 1 : 1000 dilution), anti-Bcl2 (Proteintech, Chicago, USA, 1 : 5000 dilution), anti-Bax (Proteintech, Chicago, USA, 1 : 5000 dilution), and anti-GAPDH (Proteintech, Chicago, USA, 1 : 5000 dilution) antibodies, were used to label-related proteins in this research. A chemiluminescence detection system (Millipore, Billerica, MA, USA) was used to test protein expression. These results were normalized to GAPDH and column-plotted by GraphPad Prism 7 software.

### 2.6. Quantitative (Q) Real-Time- (RT-) PCR

Total RNA was extracted from mouse specimens using TRIzol (Invitrogen, Thermo Fisher Scientific, California, USA), and the PrimeScript RT Reagent Kit (TaKaRa, Osaka, Japan) was used for synthesis of complementary DNA (cDNA) in accordance with the manufacturer's guidelines. RT-PCR was performed using an IQ5 instrument (Bio-Rad, CA, USA) by SYBR Green fluorescence signal detection assays (TaKaRa, Osaka, Japan) and primers. The -2^*ΔΔ*Ct^ method was used to analyze the expression of mRNA. The primers used in this study were as listed below: sense and antisense primers to mouse TMIGD1 (5′-GACCCGAATTCAGAAACAC-3′ and 5′-GCCCTTCTCAAAACGTA-3′) and sense and antisense primers to human TMIGD1 (5′-CTCCCATGCCATCCCTTGTTA-3′ and 5′-CGATCCTTTGCGAATGGAGAAAT-3′).

### 2.7. ROS Measurement

The changes in cellular ROS levels were tested through the following steps. First, 5 × 10^6^ mesothelial cells were inoculated on cover slips. After 24 h of cultivation, mesothelial cells were treated with H_2_O_2_ at a concentration of 500 *μ*M (Sigma Chemical Co., St. Louis, MO, USA) for 12 h. Next, after washing twice, the mesothelial cells and 10 *μ*M 2′,7′-dichlorofluorescein diacetate (DCFH-DA) were cocultured in the dark for 30 min at 37°C. Finally, a Leica microscope was used to take photos of ROS fluorescence in mesothelial cells after washing once again. The intensity was measured using ImageJ software.

### 2.8. ROS and MDA Measurement in Specimens

Frozen pathological tissue specimens were made into sections, which were stored at -80°C. ROS staining was performed using dyeing solution (catG0002, Wuhan Servicebio Technology Co., Ltd., China) according to the manufacturer's instructions, and then, the cells were cultured in DAPI dyeing solution (catG1012, Wuhan Servicebio Technology Co., Ltd., China). Sections were observed by fluorescence microscopy. The ratio of ROS-positive nuclei to total nuclei was calculated as the ROS expression level. The level of MDA was measured by a kit (Jincheng, Nanjing, China) according to the manufacturer's instructions. The results were normalized by the total protein.

### 2.9. Transmission Electron Microscopy (TEM) Analysis of Mitochondrial Morphology

A 2 mm^2^ piece of peritoneal adhesion tissue was collected and fixed in 2.5% glutaraldehyde. Then, the specimens were made into 70 nm slices and observed by TEM. We collected at least one specimen from each mouse and measured the mitochondrial length in at least three fields for one slice.

### 2.10. Survival Analysis of Mesothelial Cells

The MTT assay is an instrument for detecting the number of living cells. Approximately 5 × 10^3^ mesothelial cells were inoculated on several 96-well plates for 12 h, and then, mesothelial cells were treated with distinct concentrations of H_2_O_2_ (1000 *μ*M, 500 *μ*M, 250 *μ*M, 125 *μ*M and 62.5 *μ*M). Approximately 24 h later, the cells were soaked in 20 *μ*L MTT at a concentration of 5 mg/mL for 4 h, and the MTT crystals were soluble in DMSO. Cell viability was measured by a microplate reader (Thermo Fisher Scientific, Waltham, USA).

### 2.11. Apoptosis Analysis

Cell apoptosis was tested by a kit (Affinity BioReagents) following the instructions.

### 2.12. Mitochondrial Membrane Potential

Mitochondrial membrane potential was measured by a kit (JC-1: Solebo Biotechnology Co., Ltd.) following the instruction.

### 2.13. Scratch-Wound Assay and Adhesion Analysis

A scratch-wound assay was conducted to test the migratory potential of TMIGD1-overexpressing mesothelial cells and normal mesothelial cells. HMrSV5 cells were inoculated on 6-well plates. Cells grew to confluence and were scratched by a 200 *μ*L pipette tip. After culturing for 48 h at 37°C and 5% CO_2_, pictures were taken using a photomicroscope (Leica DFC950 camera; Leica Microsystems, Wetzlar, Germany). The cell migration ratio was quantitated by Scion Image software (beta 4.0.2, Scion, Frederick, MD). Cell adhesion was assessed by a kit (Bestbio, Beijing, China) in accordance with the instructions.

### 2.14. Statistics

Data collection and analysis were performed using SPSS 18.0 (Chicago, IL, USA) in this study. The results are presented as the percentages, absolute numbers, and the mean ± standard deviation. The *t* tests or one-way ANOVA was used to analyze normally distributed data. The differences were tested by the least significant difference method (LSD) test among the groups. The Kruskal-Wallis test was used for nonnormally distributed data analysis. The *χ*-squared test or Fisher's exact test was used for quantitative data analysis. *P* < 0.05 was considered statistically significant.

## 3. Results

### 3.1. TMIGD1 Expression Is Decreased in Mesothelial Cells

**Figure 1:**
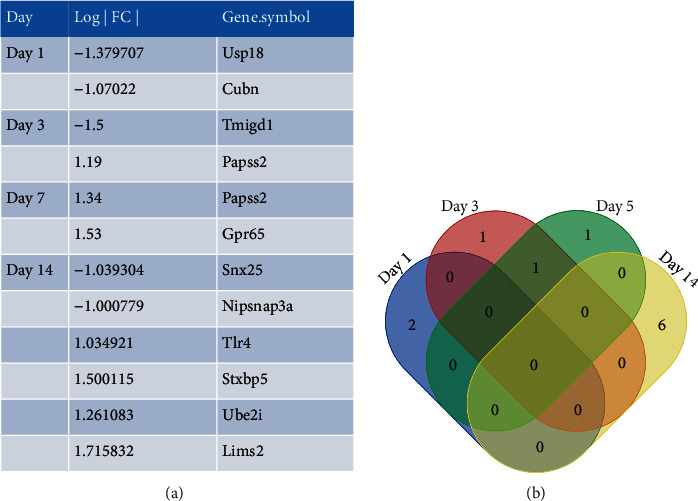
Analysis of the GEO dataset GSE4715. (a) GEO2R software showing the gene name of differentially expressed genes compared with normal peritoneum at different times, screening criteria: ∣LogFC | >1 and *P* < 0.05 (https://www.ncbi.nlm.nih.gov/geo/). (b) Venn diagram showing the coexpression of different genes in different time periods.

**Figure 2:**
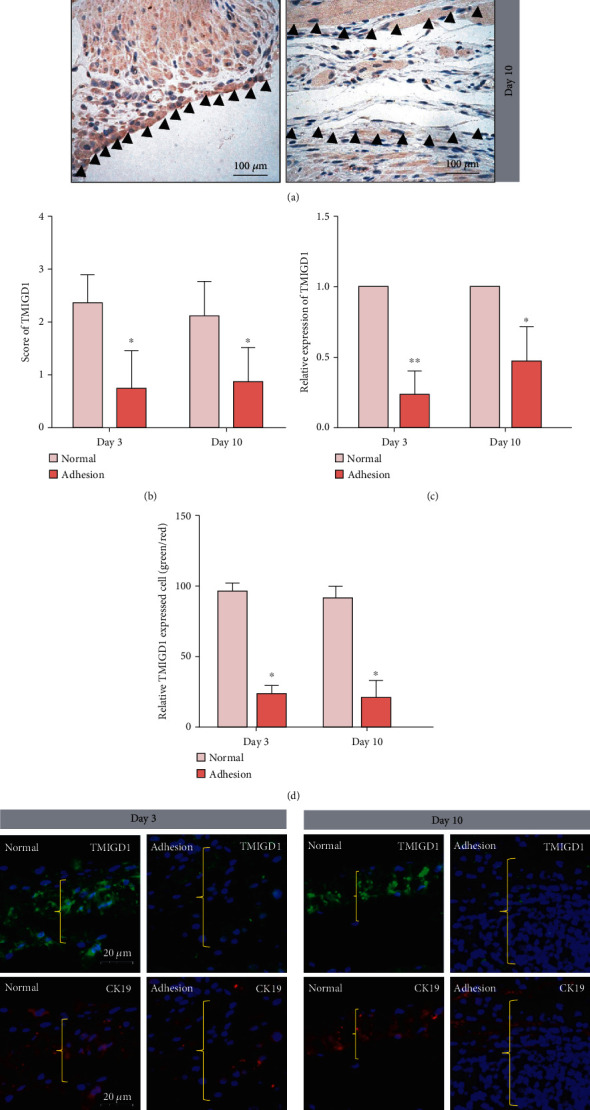
TMIGD1 expression is decreased in mesothelial cells. (a) IHC showing TMIGD1 expression in adhesion tissue and normal peritoneum of a mouse abdominal adhesion model at POD 3 and 10. *N* = 10; the black arrows mark the normal peritoneum or adhesion tissue; 400x magnification. (b) Score of TMIGD1 in the pictures of IHC of adhesion tissue and normal peritoneum of the mouse abdominal adhesion model at POD 3 and 10. *N* = 10; ^∗^compared to normal tissue, *P* < 0.05; *t* test. (c) Quantitation of qPCR for TMIGD1 shows downregulation of expression in mouse adhesion tissue (compared to the normal peritoneum of each time point) at POD 3 and 10. *N* = 10; ^∗^compared to normal tissue, *P* < 0.05; ^∗∗^compared to normal tissue, *P* < 0.01; *t* test. (d) IHF for TMIGD1 (green) and CK19 (red) in mouse adhesion tissue at POD 3 and 10. *N* = 10; the yellow brackets show normal peritoneum or adhesion tissue; 900x magnification. (e) Quantification of corresponding fluorescence intensities for TMIGD1 in mesothelial cells of IHF. *N* = 10; ^∗^compared to normal tissue, *P* < 0.05; *t* test.

We discovered that TMIGD1 expression is decreased in abdominal adhesion tissue compared to normal peritoneum at postoperative day (POD) 3 through analyzing the microarray data from the GSE4715 dataset in GEO (gene 2R software; adhesion tissue vs. normal peritoneum; screening criteria: ∣LogFC | >1 and *P* < 0.05; https://www.ncbi.nlm.nih.gov/geo/, Figures 1(a) and 1(b)). To validate this finding further, we measured TMIGD1 expression in mouse abdominal adhesion tissues via IHC. These analyses indicated that TMIGD1 expression is decreased in adhesion tissue compared to normal peritoneum at POD 3 and POD 10 (Figures 2(a) and 2(b)). q-PCR assays revealed the same molecular alteration in adhesion tissue (Figure 2(c)).

To explore the effect of TMIGD1 in mesothelial cells, we examined TMIGD1 expression in mesothelial cells of normal and adhesion tissue in noninterventional adhesion model mice at two time points (POD 3 and POD 10) using double immunofluorescence staining. The intensity of TMIGD1 (fluorescence green) was weakened in mesothelial cells of adhesion tissue at POD 3 and POD 10 (Figures 2(d) and 2(e)), contrary to normal peritoneum, in which numerous TMIGD1-expressing mesothelial cells were observed. As such, we speculate that the downregulation of TMIGD1 is a reason for mesothelial cell injury in the process of adhesion formation.

### 3.2. TMIGD1 Inhibits Abdominal Adhesion Formation

TMIGD1 expression is decreased in adhesion tissue compared with a normal control peritoneum in mice undergoing abdominal adhesion model operation. We hypothesize that TMIGD1 acts as an antiadhesion factor in the process of abdominal adhesion formation.

**Figure 3:**
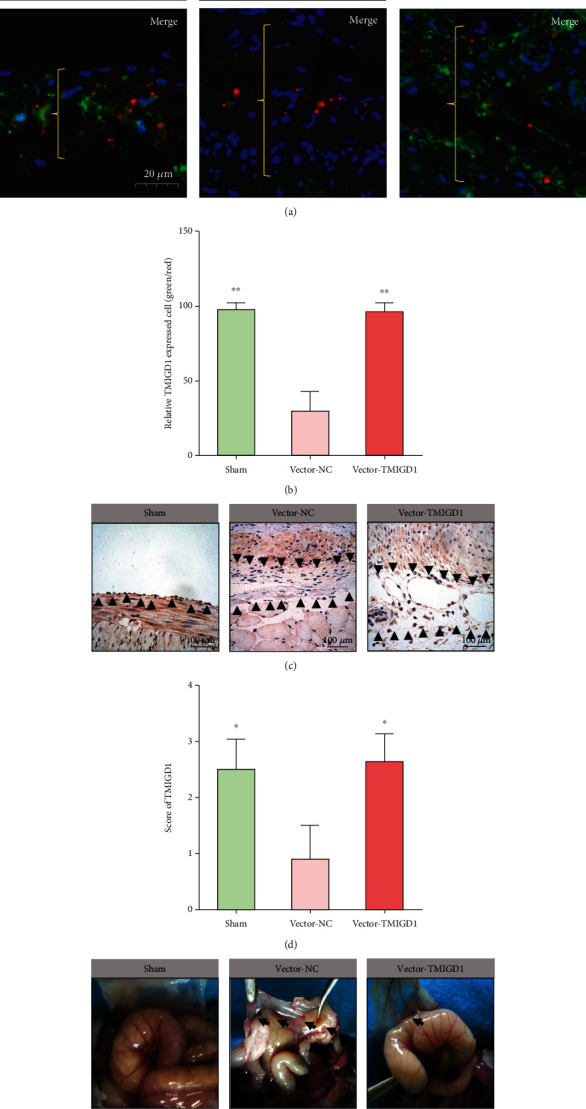
TMIGD1 inhibits abdominal adhesion formation. (a) IHF for TMIGD1 (green fluorescence) and CK19 (red fluorescence) in each group. *N* = 10; the yellow brackets show normal peritoneum or adhesion tissue; 900x magnification. (b) Quantification of relative positive TMIGD1 expression in the CK19-marked mesothelial cells in each group. *N* = 10; ^∗∗^compared to the vector-normal control (NC) group, *P* < 0.01; abnormal distribution, Kruskal-Wallis test. (c) IHC showing the expression of TMIGD1 in each group. *N* = 10; the black arrows mark the normal peritoneum or adhesion tissue; 400x magnification. (d) TMIGD1 scores in the IHC images of each group. *N* = 10; ^∗^compared to the vector-NC group, *P* < 0.05; abnormal distribution, Kruskal-Wallis test. (e) Typical gross observation of abdominal adhesion conditions in each group. *N* = 10; the black arrows mark adhesion tissue. (f) Application of an objective grade and tenacity adhesion score by two independent researchers quantifies the relative adhesion severity of each group. *N* = 10; ^∗^compared to the vector-NC group, *P* < 0.05; abnormal distribution, Kruskal-Wallis test.

To validate whether TMIGD1 might inhibit abdominal adhesion formation, we created adhesions in wild-type mice that were locally treated with lentivirus overexpressing TMIGD1 during adhesion formation, which resulted in significantly increased TMIGD1 expression in mesothelial cells of the injured peritoneum surface (Figures 3(a)–3(d)) compared to the control vector-infected mice. TMIGD1 expression resulted in significantly decreased adhesion formation compared with control vector-infected mice, which was tested by both the grade and tenacity score of adhesion (Figures 3(e) and 3(f)).

In summary, TMIGD1 acts as an anti-adhesion factor in the process of abdominal adhesion formation. Low TMIGD1 expression increased postoperative abdominal adhesion formation.

### 3.3. TMIGD1 Protects Mesothelial Cells from Oxidative Stress Injury

**Figure 4:**
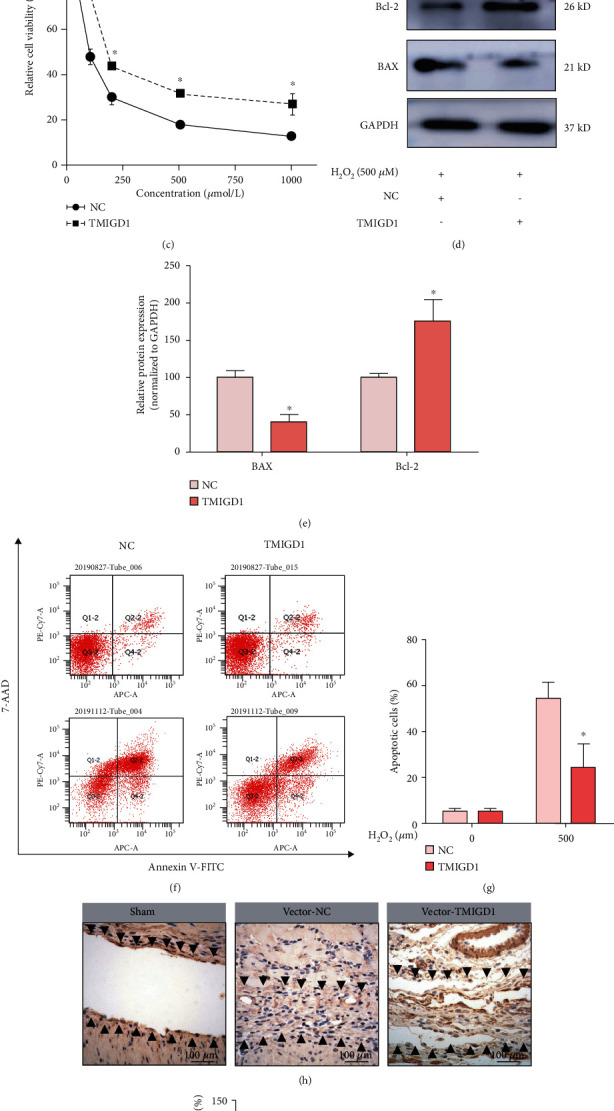
TMIGD1 protects mesothelial cells from oxidative stress injury. (a) qPCR identified TMIGD1 expression in the NC and TMIGD1-overexpressing cell lines. *N* = 3; ^∗∗^compared to the NC, *P* < 0.01, *t* test. (b) Western blotting identified TMIGD1 expression in the NC and TMIGD1-overexpressing cell lines; *N* = 3. (c) MTT assay showing the viability of mesothelial cells undergoing oxidative stress injury induced by different concentrations of H_2_O_2_ in the NC and TMIGD1-overexpressing cell lines. *N* = 5; ^∗^compared to the NC, *P* < 0.05; *t* test. (d) Western blotting showing the apoptosis-related protein expression of mesothelial cells undergoing oxidative stress injury induced by H_2_O_2_. *N* = 3. (e) Quantification of the apoptosis-related protein expression of mesothelial cells undergoing oxidative stress injury induced by H_2_O_2_. ^∗^Compared to the NC, *P* < 0.05; *t* test. (f) The apoptosis levels of mesothelial cells examined by flow cytometry transfected with a vector of NC or TMIGD1 overexpression and treated with H_2_O_2_ for 24 hours (mean ± SD). *N* = 3. (g) Quantification of the apoptosis levels of mesothelial cells examined by flow cytometry. ^∗^Compared to the NC treated with 500 *μ*M of H_2_O_2_, *P* < 0.05; *t* test. (h) CK19 staining showing the severity of mesothelial cell injury in each group. *N* = 10; the black arrows mark the normal peritoneum or adhesion tissue; 400x magnification. (i) The completeness rate of peritoneal mesothelial cells in each group. *N* = 10; ^∗^compared to the vector-NC group, *P* < 0.05; abnormal distribution, Kruskal-Wallis test.

To determine whether TMIGD1 protects mesothelial cells from H_2_O_2_-induced cell injury, we constructed HMrSV5 cell lines that stably express TMIGD1 (Figures 4(a) and 4(b)) and treated both the control cell line and TMIGD1-overexpressing cell line with different concentrations of H_2_O_2_. Subsequently, an MTT assay was used to measure cell viability, and we found that the expression of TMIGD1 increased the survival of mesothelial cells in response to cell injury (Figure 4(c)). Then, the expression of apoptosis-associated proteins such as Bax and Bcl-2 and Annexin V apoptosis assays was used to assess mesothelial cell apoptosis. Bax expression was significantly decreased; however, Bcl-2 expression was significantly increased in the TMIGD1-overexpression group compared with the control group (Figures 4(d) and 4(e)). Similarly, Annexin V apoptosis analysis suggested that the ratio of apoptotic cells in the TMIGD1-overexpression group was significantly decreased compared with that in the control group (Figures 4(f) and 4(g)), which was quantified by flow cytometry. This finding was further validated in vivo. CK19 staining of mouse peritoneal specimens at POD 10 showed that the TMIGD1-overexpression group had a higher mesothelial cell completeness rate than the vector control group (Figures 4(h) and 4(i)).

Taken together, these data suggested that the presence of TMIGD1 protects mesothelial cells from hydrogen peroxide-induced oxidative stress injury.

### 3.4. TMIGD1 Alleviates Oxidative Stress by Protecting the Mitochondrial Function of Mesothelial Cells

**Figure 5:**
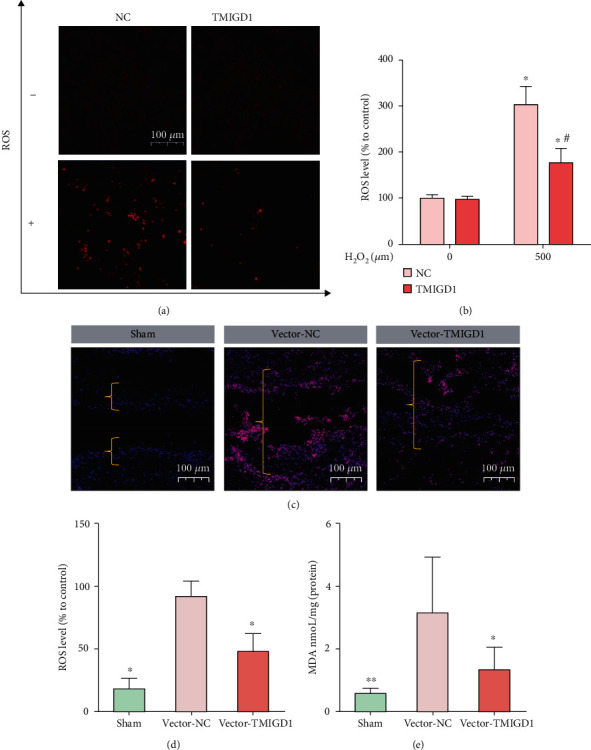
TMIGD1 alleviates oxidative stress in the mesothelial cells. (a) ROS levels in mesothelial cells transfected with a vector of NC or TMIGD1 overexpression and treated with H_2_O_2_ (mean ± SD). *N* = 3; 200x magnification. (b) Quantification of ROS levels in mesothelial cells transfected with a vector of NC or TMIGD1 overexpression and treated with H_2_O_2_. ^∗^Compared to mesothelial cells without the induction of H_2_O_2_, *P* < 0.05; ^#^compared to the NC group with the induction of 500 *μ*M of H_2_O_2_, *P* < 0.05; *t* test. (c) IHF showing the ROS intensity in each group; the yellow brackets show normal peritoneum or adhesion tissue. *N* = 5; 400x magnification. (d) Quantification of relative ROS intensity in each group. *N* = 5; ^∗^compared to the vector-NC group, *P* < 0.05; abnormal distribution, Kruskal-Wallis test. (e) The MDA level in each group. *N* = 10; ^∗^compared to the vector-NC group, *P* < 0.05; ^∗∗^compared to the vector-NC group, *P* < 0.01; abnormal distribution, Kruskal-Wallis test.

To verify that TMIGD1 protects mesothelial cells from injury by alleviating oxidative stress, we measured the ROS level in the different TMIGD1-expressing and H_2_O_2_-treated groups and found that the ROS level was significantly decreased in the TMIGD1-overexpressing group compared to the control group under the induction of hydrogen peroxide (Figures 5(a) and 5(b)). IHF demonstrated that the sham operation group and the TMIGD1-overexpression group had lower levels of ROS in the adhesion tissue of mice than the control vector group (Figures 5(c) and 5(d)). In addition, we detected the oxidative stress injury-relevant marker MDA in normal peritoneal or adhesion tissue specimens and found that MDA was significantly higher in the vector control group than in the sham group, but significantly lower in the TMIGD1 overexpression group than in the vector control group (Figure 5(e)).

**Figure 6:**
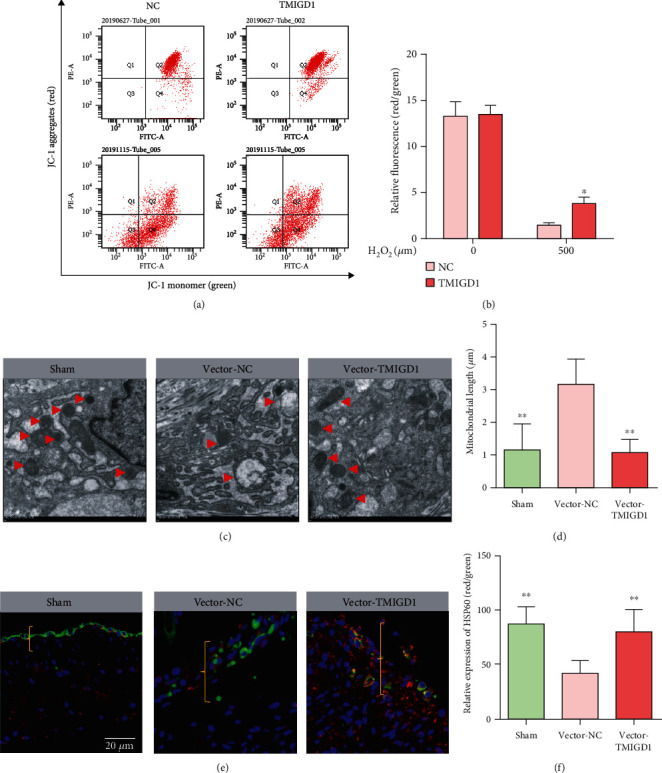
TMIGD1 alleviates oxidative stress by protecting the mitochondrial function of mesothelial cells. (a) JC-1 staining showing the mitochondrial membrane potential level of mesothelial cells. The flow cytometry scatter plot showing the distribution of cell populations with JC-1 aggregates (red) and JC-1 monomers (green); *N* = 3. (b) Histogram of the relative ratio of red to green fluorescence (mean ± SD). *N* = 3; ^∗^compared to the NC with the induction of H_2_O_2_, *P* < 0.05; *t* test. (c) Transmission electron microscopy scan of mitochondrial conditions in each group. *N* = 3; the red arrow marks mitochondria, 10000x magnification. (d) The mitochondrial length in each group. *N* = 3; ^∗∗^compared to the vector-NC, *P* < 0.01; abnormal distribution, Kruskal-Wallis test. (e) IHF staining of mitochondria in each group. *N* = 10; the red marked HSP60, the green marked the CK19, and the blue marked the DAPI; the yellow brackets show normal peritoneum or adhesion tissue; 10000x magnification. (f) Quantification of relative HSP60 expression in each group of (e). *N* = 3; ^∗∗^compared to the vector-NC, *P* < 0.01; abnormal distribution, Kruskal-Wallis test.

Since mitochondria is a major source of ROS, the increase of ROS may indicate mitochondrial dysfunction [12]. It has been reported that TMIGD1 interacts with the mitochondrial outer membrane protein SYNJ2BP and can be recruited to mitochondria [20]. In view of the above exploration, we hypothesized that TMIGD1 alleviates oxidative stress by protecting the mitochondrial function of mesothelial cells. To zero in on the role of TMIGD1 in mesothelial cells, we measured the mitochondrial membrane potential level of mesothelial cells from the TMIGD1-overexpression group and the control group of HMrSV5 cell lines by JC-1 staining that was treated with H_2_O_2_; results displayed that the mitochondrial membrane potential was significantly decreased in the mesothelial cells from the control group compared to the TMIGD1-overexpression group (Figures 6(a) and 6(b)). We also used TEM to assess any mitochondrial alterations of mesothelial cells in the peritoneal tissue specimens and found that mitochondria in the sham-operation group appeared to be small elliptic/circular high electron-dense structures with clear and compact mitochondrial crista in TEM 2D sections. In the TEM section of the control vector group, there were a number of round/swollen structures present whose volume enlarged and electron density decreased, with mitochondrial crista of cytoplasm arefaction or destruction. However, in the TMIGD1-overexpression group, the swelling of mitochondria structures lighten, the volume of mitochondria structures decreased, and the electron density increased compared to the control vector group. Mitochondrial morphologies similar to those of the sham-operation group constitute the majority (Figures 6(c) and 6(d)). In brief, it was shown that the TMIGD1-overexpression group had a significantly more regular mitochondrial morphology than the control vector group, which strikingly contributed to normal function.

To further verify our result, we performed IHF to detect the HSP60 (a mitochondrial chaperonin, marked mitochondria with normal structure and function) [21] in CK19-marked mesothelial cells and found that HSP60 was expressed higher in the sham and TMIGD1 overexpression group when compared to the control group (Figures 6(e) and 6(f)), suggesting that TMIGD1 overexpression reduced the number of damaged mitochondria by ROS in adhesion formation.

Apparently, our findings provide insight into a new possible mechanism through which TMIGD1 can exert its function in preventing mesothelial cells from oxidative stress.

### 3.5. TMIGD1 Promotes Cell Adhesion between Mesothelial Cells

Cell adhesion plays a main role in cell injury and healing. TMIGD1 was predicted to act as a cell adhesion molecule in some previous studies.

**Figure 7:**
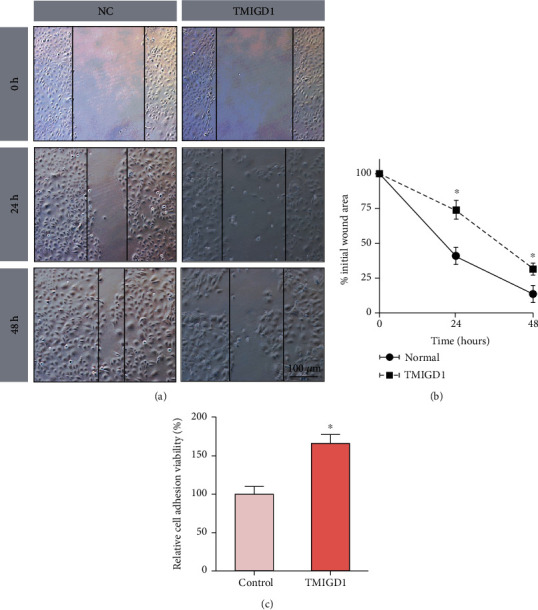
TMIGD1 promotes cell adhesion between mesothelial cells. (a) The cell scratch assay showing the ratio of cell migration in the NC and TMIGD1-overexpressing cell lines at different time points. *N* = 5; 100x magnification. (b) Quantitation of the wound scratch healing rate of NC- and TMIGD1-overexpressing cell lines at different time points. *N* = 5; ^∗^compared to the NC, *P* < 0.05; *t* test. (c) Cell adhesion viability in the NC and TMIGD1-overexpressing cell lines. *N* = 5; ^∗^compared to the NC, *P* < 0.05; *t* test.

To determine whether TMIGD1 promotes mesothelial cell adhesion, a cell scratch assay was performed. The migration rate of mesothelial cells of the TMIGD1-overexpressing cell line was significantly decreased compared with that of the control cell line (Figures 7(a) and 7(b)). To directly prove this, we assessed the adhesive capability of mesothelial cells and found that cell adhesion was enhanced between mesothelial cells of the TMIGD1-overexpressing cell line (Figure 7(c)). Taken together, TMIGD1 promotes cell adhesion between mesothelial cells.

## 4. Discussion

The peritoneum is a double-layered tissue that lines in the abdominal cavity and the surface of intra-abdominal organs, consisting of a continuous mesothelial monolayer and underlying connective tissue [22]. Abdominal adhesion formation is a process of damaged peritoneum healing [6], in which mesothelial cell healing plays a considerable role. Under inflammatory conditions, mesothelial cells undergo apoptosis, die, or transform into mesenchymal cells, all of which result in further damage to the peritoneum [7]. Thus, elucidating the underlying mechanism of mesothelial cell injury and healing is extremely essential to understand how postoperative abdominal adhesion forms. In this study, we showed that TMIGD1 protects mesothelial cells from H_2_O_2_-induced oxidative stress injury by protecting mitochondria. However, TMIGD1 expression is decreased in abdominal adhesion tissue. In addition, TMIGD1 promotes cell adhesion.

Oxidative stress is a regular process that occurs in the healing of a damaged peritoneum [23]. However, little is known about the molecular mechanism of oxidative stress injury of abdominal adhesion [11]. The peritoneal tissue microenvironment is proinflammatory after damage. Necrotic cells, clots, and recruited inflammatory cells release a large amount of ROS [24]. Although a certain concentration of ROS is beneficial for tissue healing, a large amount of ROS damages peritoneal mesothelial cells, which leads to mesothelial cell death and apoptosis and contributes to the transformation of mesothelial cells into mesenchymal cells [25, 26]. We have demonstrated that TMIGD1 overexpression reduces ROS levels in mesothelial cells both in vitro and in vivo, which is useful to prevent ROS injury in mesothelial cells. In some previous studies, TMIGD1 was considered an ROS scavenger gene in kidney epithelial cells [14]; here, we showed that it has an identical effect in mesothelial cells. However, ROS produced by mesothelial cells count only a part of ROS produced in the inflammatory environment of adhesion. Follow-up experiments are needed to explore other specific mechanisms by which TMIGD1 reduces ROS levels in adhesive environments.

After injury or trauma, ischemia and hypoxia lead to the accumulation of large amounts of ROS. Under this ischemic condition, the source of ROS in cells mainly comes from remodeled mitochondrial electron transport chains (approximately 70% of ROS come from mitochondria); thus, protecting mitochondrial function is very important for reducing ROS [13]. It has been reported that TMIGD1 can localize to mitochondria [20]. Here, we demonstrated that TMIGD1-overexpressing mesothelial cells have significantly more normal mitochondria, suggesting that TMIGD1 inhibits mesothelial cell mitochondrial injury induced by ROS, which might be our new findings and partly illustrate the possible mechanism by which TMIGD1 reduces ROS.

The results presented here demonstrated that TMIGD1 promotes cell adhesion. Under the influence of inflammation, the injured mesothelial cells around the injured peritoneal area may be shed from the peritoneal tissue and die. Thus, promoting cell adhesion could modify the poor state and promote cells floating in the abdominal cavity to adhere to the peritoneal tissue, which may be beneficial to peritoneum healing [27]. Mesothelial cell healing of the peritoneum may originate from the migration of proliferating mesothelial cells at the edge of the incision, stem cells, transformation of macrophages, exfoliation and implantation of free-floating serosal cells in the abdominal cavity [4]. Promoting cell adhesion may be another mechanism by which TMIGD1 participates in peritoneal healing.

The limitation of this study is that we did not explore these results in human abdominal adhesion because of the extremely low expression of TMIGD1 in other human tissues except renal tissues. However, we must not exclude the possibility that local administration of TMIGD1 drugs may be effective for the prevention of adhesion formation. Our findings likely declare the possible mechanism of abdominal adhesion formation that contributes to the treatment and prevention of postoperative abdominal adhesion formation.

## 5. Conclusion

In conclusion, TMIGD1 protects mesothelial cells from oxidative stress injury by protecting their mitochondrial function, which is decreased in regular abdominal adhesion tissue. In addition, TMIGD1 enhances peritoneal mesothelial cell adhesion to promote healing.

## Data Availability

The data used to support the findings of this study are available from the corresponding author upon request.
